# Rapid and sustained differentiation of disease-suppressive phyllosphere microbiomes in tomato following experimental microbiome selection

**DOI:** 10.1186/s40793-025-00734-1

**Published:** 2025-07-01

**Authors:** Hanareia Ehau-Taumaunu, Terrence H. Bell, Javad Sadeghi, Kevin L. Hockett

**Affiliations:** 1 Department of Plant Pathology and Environmental Microbiology, The Pennsylvania State University, University Park, PA 16802 USA; 2 Department of Physical and Environmental Sciences, University of Toronto – Scarborough, Toronto, ON Canada; 3 One Health Microbiome Center, Huck Institutes of the Life Sciences, The Pennsylvania State University, University Park, PA 16802 USA

**Keywords:** *Pseudomonas syringae*, Tomato, Disease suppression, Bacterial speck, Microbiome

## Abstract

**Background:**

Microbial-based treatments to protect plants against phytopathogens typically focus on soil-borne disease or the aboveground application of one or a few biocontrol microorganisms. However, diverse microbiomes may provide unique benefits to phytoprotection in the phyllosphere, by restricting pathogen access to niche space and/or through multiple forms of direct antagonism. We previously showed that successive experimental passaging of phyllosphere microbiomes along with the phytopathogen *Pseudomonas syringae* pv. *tomato* (*Pto*), which causes bacterial speck in tomato, led to the development of a disease suppressive microbial community. Here, we used amplicon sequencing to assess bacterial and fungal composition at the end of each passage, as well as shotgun metagenomics at key passages based on observed disease-suppressive phenotypes, to assess differences in functional potential between suppressive and non-suppressive communities.

**Results:**

Bacterial composition changed and diversity declined quickly due to passaging and remained low, particularly in treatments with *Pto* present, whereas fungal diversity did not. *Pseudomonas* and *Xanthomonas* populations were particularily enriched in disease-suppressive microbiomes compared to conducive microbiomes. The relative abundance of *Pseudomonas syringae* group gemonosp. 3 (the clade to which the introduced pathogen belongs) in shotgun metagenomic data was similar to what we observed for *Pseudomonas* ASVs in the 16S rRNA gene dataset. We also observed an increase in the abundance of genes associated with microbial antagonism at Passage 4, corresponding to the highest observed disease severity.

**Conclusions:**

Taxonomic richness and evenness were low within samples, with clustering occurring for suppressive or non-suppressive microbiomes. The relative abundance of genes associated with antagonism was higher for disease-suppressive phyllosphere microbiomes. This work is an important step towards understanding the microbe-microbe interactions within disease-suppressive phyllosphere communities.

**Supplementary Information:**

The online version contains supplementary material available at 10.1186/s40793-025-00734-1.

## Background

Plant-associated microbiomes play critical roles in maintaining plant health and suppressing plant diseases caused by foliar phytopathogens [1–4]. As a result, there is interest in manipulating these microbiomes to better manage plant health to meet our agricultural objectives. Previous work has attempted to shift plant-associated microbiomes to provide disease suppression by introducing exogenous microorganisms, but the use of single microbial agents has seen limited success in protecting plants [5, 6]. Broadening our approach to foliar disease management to include multi-strain/multi-organism consortia or whole microbiomes could be advantageous, as this could allow multiple taxa to fill available leaf space and/or to incorporate diverse and additive forms of antagonism [7]. Assessment of naturally-occurring and synthetic microbiomes that suppress disease has enabled researchers to gain insight into how microbiomes respond to phytopathogens and how we might manipulate microbiomes to induce disease suppression [8–11]. Many of these studies remain focused on rhizosphere and soil microbiomes, while few have characterized microbiomes that are capable of disease suppression in the phyllosphere [12–15].

Numerous studies have assessed microbial composition and microbe-microbe interactions in the rhizosphere in the context of disease-suppressive soils, in which disease incidence or severity remains low in susceptible plants, even in the presence of a virulent pathogen [16–20]. When comparing the microbiomes of soils containing *Rhizoctonia solani*, Mendes et al. [21] showed that *Pseudomonadaceae, Burkholderiaceae, Xanthomonadales*, and *Lactobacillaceae* were disproportionately abundant in soils shown to be suppressive to *R. solani* relative to those conducive to *R. solani* infection. Antifungal activity has been shown in soils suppressive to *R. solani* by members of the *Pseudomonadaceae* that can produce a putative chlorinated lipopeptide, and *Streptomyces* species and *Paraburkholderia graminis,* which were profiled to produce volatile organic compounds [22, 23]. In wheat, Take-all decline, a well studied example of disease suppression, is attributed to fluorescent *Pseudomonas* spp. that exhibit antagonism towards *G. graminis* via the production of antimicrobial secondary metabolites like phenazines and the polyketide 2,4-diacetylphloroglucinol [18, 24, 25]. Other bacterial populations have also been associated with Take-all decline soils, such as *Acidobacteria*, *Planctomycetes*, *Nitrospira*, *Chloroflexi*, *Alphaproteobacteria*, and *Firmicutes* [26].

Despite a body of research into the microorganisms and associated functions in disease-suppressive soils, similar investigations of disease-suppressive *phyllosphere* microbiomes are sparse. This is likely due to the transient nature of phyllosphere microbes and many current cultural management practices that reduce the ability of phyllosphere microbiomes to develop long-term evolutionary and ecological trajectories that respond to a given pathogen. Investigations into the functional potential of phyllosphere microorganisms have uncovered several mechanisms of microbial antagonism that are also observed in suppressive soils, such as resource competition, production of antimicrobials, and activation of plant defenses against potential pathogens [1, 27–29]. Secondary metabolites encoded by biosynthetic gene clusters, which can have antimicrobial activity, have been identified in many phyllosphere bacteria, including non-ribosomal peptide synthases, polyketide synthases, and ribosomally-synthesized and post-translationally modified peptides [30–32]. The production of antibiotics is also known to modulate the diversity and abundance of microbes, and many phyllosphere microorganisms can contain antibiotic resistance genes [30, 33, 34]. Other general examples of functions related to disease suppression are chitinases, terpenes, and terpenoids, which can act against phytopathogens [35–37].

We previously showed that experimentally passaging phyllosphere microbiomes alongside the bacterial pathogen *Pseudomonas syringae* pv. *tomato* (*Pto*), which causes bacterial speck in tomato, results in the development of disease-suppressive phyllosphere microbiomes [38]. During this study, bacterial speck disease severity increased over the first ~ 4 passages, followed by a decline in subsequent passages, resulting in low disease severity by the end of the experiment. Importantly, disease suppression only developed for communities in which the pathogen was included in the passaging process and was lost by autoclaving of the suppressive community. Therefore, under the right ecological conditions, disease suppression could be developed, and that suppression was apparently related to microbial activity. In this study, using a combination of amplicon and shotgun metagenomic approaches, we assessed the composition and functional potential of the passaged phyllosphere microbiomes from our previous study that were observed to be either suppressive or non-suppressive toward *Pto*. We hypothesized that (1) microbiome composition would differentiate between suppressive and non-suppressive communities due to continued interaction with the pathogen and (2) the relative abundance of genes potentially related to antagonism would be higher within disease-suppressive microbiomes. These analyses give insight into how disease-suppressive phyllosphere microbiomes developed in our study and what taxa and functions may be required to promote suppression in the field.

## Methods

### Passaging experiment, microbial collection, and DNA extraction

**Fig. 1:**
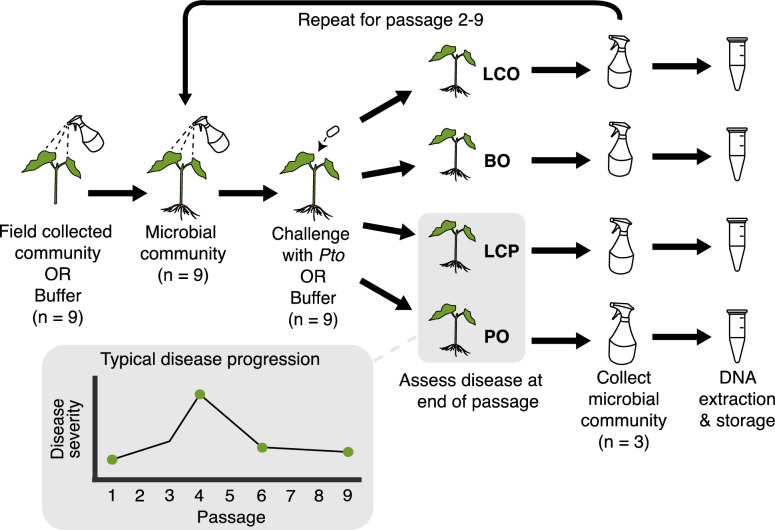
Overview of experimental design for a single passage line for collecting DNA from Ehau-Taumaunu and Hockett [38] for this study. Individual samples were collected from each treatment of four passage lines at all passages for amplicon sequencing and key passages (1, 4, 6, and 9) for shotgun metagenomic sequencing (shown in the grey box). Nine passages of the passaging experiment were completed for four passage lines. Treatments are LCO = leaf community only, BO = buffer only, LCP = leaf community with *Pto*, and PO = pathogen only

The samples used in this study came from the previous work of Ehau-Taumaunu and Hockett [38], which showed the development of disease-suppressive microbiome phenotypes across 9 experimental passages. Briefly, to begin the first passage, 1.5-week-old tomato plants were sprayed with an exogenous phyllosphere microbiome (n = 18) or MgCl_2_ buffer (n = 18; Fig. 1). Two days later, each set of plants was sprayed either with the phytopathogen *Pseudomonas syringae* pv. *tomato* (*Pto*) or MgCl_2_ buffer. Four independent treatments consisting of 9 replicate tomato plants were produced: *leaf community and Pto*’ (LCP), ‘*leaf community only*’ (LCO), ‘*Pto only*’ (PO), and ‘*buffer only*’ (BO). Five days later, the disease severity (i.e., percentage of leaf surface area that appeared diseased) of all plants was assessed. The three tomato plants of LCP and PO treatments exhibiting the least disease were selected for transfer to the next passage. For LCO and BO treatments, three plants were selected at random due to the apparent absence of disease. The three plants from each treatment were the source of phyllosphere microbiomes sprayed onto new tomato plants (n = 9 for each treatment) to begin the next passage. A total of nine passages were conducted with four passage lines (107, 108, 109, and 110). At the end of each passage, 500 mL of each microbiome was filtered through a 0.22 uM polyethersulfone membrane filter (VWR, Randor, PA, USA) and stored at – 80 °C. The microbial DNA was extracted from the filters using a phenol–chloroform protocol [38]. Total DNA was quantified using a Qubit 2.0 Fluorometer and the Qubit dsDNA HS assay (Invitrogen, Waltham, MA, USA). All samples were stored at -20˚C.

### Amplicon and shotgun sequencing workflows

The preparation of 16S rRNA gene and ITS1 region amplicon libraries for all samples collected for each treatment across all nine passages of the four biological replicates (n = 148) followed a procedure similar to Trexler and Bell [39]. Briefly, initial amplification was performed with the 515F/806R universal 16S rRNA gene primers and Internal transcribed spacer 1 (ITS1) region primers ITS1F/58A2R, with all primers modified to include standard Illumina overhang sequences for barcode attachment [40–43]. Amplicons were prepared in triplicate, and two negative controls were included for both bacteria and fungi. Both libraries were cleaned, indexed, and normalized. Libraries were separately pooled for 16S rRNA gene and ITS amplicons, concentrated using a Savant SpeedVac (Thermo Fisher Scientific, Waltham, MA, USA), and purified. Sequencing was performed at the Huck Institutes of the Life Sciences Genomics Core Facility at The Pennsylvania State University on two lanes of Illumina MiSeq using the 2 × 250 cycle v2 kit.

Shotgun metagenomic sequencing consisted of samples from passages 1, 4, 6, and 9 of the ‘*leaf community and Pto*’ (LCP) and ‘*leaf community only*’ (LCO) treatments from all biological replicates (n = 31; except for 108 LCP from passage 1). These treatments were selected to examine the effects of an exogenous field-collected phyllosphere community and the passages were selected as critical stages in plant disease progression in this experiment. The library was generated with the Illumina DNA PCR-Free Library Prep Kit and sequenced on a NextSeq 550 with a Mid-Output kit to generate 150 bp paired-end read lengths at the Huck Institutes of the Life Sciences Genomics Core Facility. All raw sequence data for amplicon and shotgun metagenomic sequencing is available at the NCBI Sequence Read Archive database under BioProject ID PRJNA932145.

### Amplicon data processing and analysis

Initial sequence processing of all 148 samples followed the recommended DADA2 pipeline (v1.18) in R (v4.0.5) for both 16S rRNA gene and fungal ITS reads [44]. Taxonomy was assigned using Silva nr99 (v138.1) for 16S rRNA gene sequences and UNITE (v8.2) for ITS sequences [45–47]. All downstream analyses were performed in R (v4.0.5), with ASVs with less than two reads or not classified at the phylum level removed from 16S rRNA gene and ITS datasets. Additionally, ASVs classified as chloroplast or mitochondria were removed. Three ITS samples were omitted due to low read counts after filtering. No normalization or subsampling of reads was performed unless specified [48]. Packages phyloseq (v1.36.0), vegan (v2.5–7), and ggplot2 (v3.3.5) were used to explore bacterial and fungal composition and diversity indexes [49–51].

Shannon index was calculated using the ‘estimate_richness’ function in phyloseq with Kruskal–Wallis and the pairwise Wilcoxon rank-sum post-hoc tests used to test passage and treatment effects. Principle Coordinate Analysis (PCoA) was also performed on normalized data in phyloseq with Bray–Curtis dissimilarities to test beta diversity. PERMANOVA analyses with 999 permutations were performed on the Bray–Curtis dissimilarities between normalized samples using the ‘adonis2’ and ‘pairwise.adonis2’ functions in vegan. P-value adjustment was conducted using a false discovery rate (FDR) algorithm with an adjusted P ≤ 0.05 cut-off. Differentially abundant taxa were defined as those identified in two or more methods (DESeq2; ANCOM; Corncob; Limma Voom) with significance defined as *P* ≤ 0.05 and Benjamini–Hochberg-adjusted *P*(FDR) ≤ 0.05 [52–56].

### Shotgun metagenomic data processing and analysis

Raw sequence data for the 31 samples were quality filtered and evaluated using FastQC (v0.11.7). Adaptors and low-quality bases were removed with Trimmomatic (v0.39) using the following parameters: PE -phred33 ILLUMINACLIP:PCRfreePE-PE.fa:2:30:10 LEADING:3 TRAILING:3 SLIDINGWINDOW:4:15 MINLEN:50 HEADCROP:14 [57]. At the microbiome level, metaSPAdes (v3.13.0) was used to co-assemble reads of all samples of the same treatment (‘leaf community with *Pto* (LCP)’ or ‘leaf community only (LCO)’) from the four passage lines [58]. Taxonomic classification and relative abundance estimation were done using Kraken 2 with the standard reference database (https://benlangmead.github.io/aws-indexes/k2) [59]. Sequences were cleaned of tomato (*Solanum lycopersicum*), human (hg37dec_v0.1), and ribosomal RNA gene (SILVA_128_LSUParc_SSUParc_ribosomal_RNA) contaminating sequences using the KneadData pipeline (https://github.com/biobakery/kneaddata). Viral reads were predicted in KBase using VirSorter2 (v2.2.3), with gene prediction performed in Prokka (v1.14.5), and taxonomy assigned using vCONTACT2 (v0.9.19) [60–63]. Additionally, two subsets of data were formed for downstream analyses based on taxonomy: (1) all contigs identified to the *Pseudomonas* genus were removed, and (2) all *Pseudomonas syringae* classified and unclassified contigs were removed. The subset datasets were to compare the effects of *Pseudomonas* or *Pseudomonas syringae* on the microbiomes.

At the metagenome-assembled genome (MAG) level, individual-based assembly of all samples was performed by metaSPAdes (v3.13.0). Contigs were binned with metaWRAP (v1.3) using MaxBin2 (2.2.5), metaBAT2 (2.12.1), and CONCOCT (1.1.0) metagenomic binning software [64–67]. The resulting bins were refined and reassembled into MAGs using metaWRAP. The MAGs underwent manual refinement using the Anvi’o (v7.1) metagenomic workflow to remove contamination and split MAGs based on coverage [68]. The MAGs produced by Anvi’o were then dereplicated (identifying similar genomes and selecting the optimal genome) across the treatments of the four passage lines with dRep (v2.4.0) using a 95% average nucleotide identity (ANI) threshold to get the complete set of MAGs [69]. The quality of MAGs was assessed with CheckM (v1.1.3) and taxonomically classified using GTDB-tk (2.0.0) based on the database release207 [70, 71]. Dereplication of MAGs across all communities was performed using dRep based on ANI.

The contig abundance of microbiomes and MAGs was determined by mapping reads back to the assembled contigs using minimap2 (v2.21) and samtools (v1.9) [72, 73]. Following mapping, normalised relative gene abundance was estimated from read alignments using CoverM (v0.6.1) and expressed as TPM (transcripts per million) [74]. The relative gene abundance was considered equivalent to the abundance of the contig in which it was encoded. The TPM of genes that were classified to the same functional category were subsequently summed. Microbial gene prediction of co-assembled microbiomes and MAGs was performed by Prodigal (v2.6.3), and functional annotations were obtained using EggNOG-mapper v2 against the eggNOG v5 database with default settings to obtain identifiers from the Pfam protein domain database [75–77]. Functional genes linked to microbial antagonism that received annotations were selected from Pfam IDs and were summed together under broad functional categories (Table S4). Heatmaps were produced in R (v4.0.5) using ‘heat.clust’ and ‘heatmap.2’ functions in massageR (0.7.2) and gplots (3.1.3), respectively. Differences in relative abundance of each treatment for a passage were tested in one-way ANOVA for each dataset with Tukey HSD pairwise comparisons in R.

## Results

### Microbiome composition was dominated by a small number of bacterial and fungal genera across experimental passages

Microbiome samples were recovered at the end of each passage (i.e., 1–9) and sequenced to examine how phyllosphere bacterial and fungal composition changed as disease suppression developed (Fig. 1). After filtering, amplicon reads were sorted into 264 bacterial and 2590 fungal ASVs (Table S1). The unusualy high number of fungal ASVs is due to a large number of ASVs with reads in only one to a few samples. This likely due to many low abundant taxa that do not become dominant across passages and that the greenhouse environment provided a greater abundance of fungi seeding the plants compared to bacteria. Thus, the bacteria:fungi ratio is skewed and we expect fungal patterns within and across passages to be less meaningful to what we observe for bacteria. Additionally, due to the low number of sequences and simple taxonomic diversity of the microbiomes, rarefaction did not change results and was omitted for the analyses.

Overall, Proteobacteria, Ascomycota, and Basidiomycota were the dominant phyla. The exogenous field-collected phyllosphere communities (i.e., Passage 0) sprayed onto the leaf community with *Pto* (LCP) and leaf community only (LCO) plants included a high relative abundance of *Sphingomonas*, *Methylobacterium*-*Methylorubrum*, and *Papiliotrema* (Figure S1). By the end of Passage 1, the dominant bacterial genera shifted to *Pseudomonas* (49 ASVs) and *Xanthomonas* (14 ASVs) regardless of treatment and passage line. As the two dominant bacterial taxa were of similar abundance for treatments that were inoculated with *Pto* and for those that were not, analyses were combined for LCP and pathogen only (PO) treatments as well as LCO and buffer only (BO), known as ‘treatments with *Pto*’ and ‘treatments without *Pto*’ respectively.

**Fig. 2:**
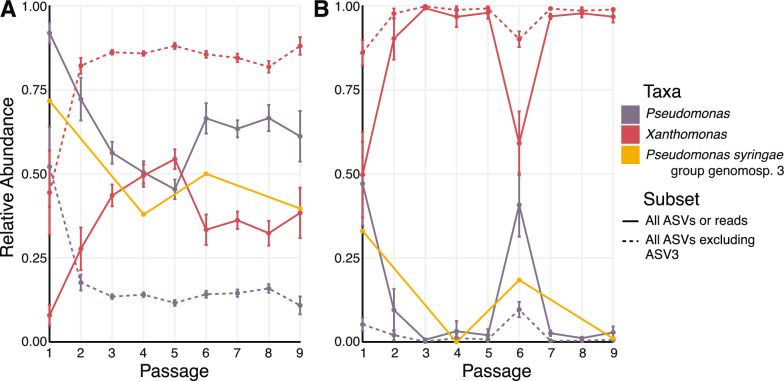
Bacterial composition (both amplicon and metagenomics data) across nine passages. The relative abundance of *Pseudomonas*, *Xanthomonas*, and *Pseudomonas syringae* group genomosp. 3 with or without ASV3 was measured for (**A**) treatments with *Pto* (LCP and PO) and (**B**) treatments without *Pto* (LCO and BO)

*Pseudomonas* begins at a high relative abundance in treatments with *Pto*, yet declines to ~ 45% at Passage 5 before increasing to ~ 60% for Passage 6 to 9 (Fig. 2A). Conversely, *Xanthomonas* mirrors the relative abundance of *Pseudomonas* by increasing and then decreasing in relative abundance across passages, whereas for treatments without *Pto*, *Xanthomas* was dominant across all passages except for a spike in *Pseudomonas* abundance at Passage 1 and 6 (Fig. 2B). ASV3 was the only ASV identified as *Pseudomonas syringae*. The removal of ASV3 from all treatments resulted in a decrease in the relative abundance of *Pseudomonas,* indicating *Pseudomonas syringae* dominated bacterial composition (Fig. 2A, B). The relative abundance of fungi shifted to *Alternaria* and *Cladosporium* from Passage 1 onwards, which were at low abundance in the exogenous microbiomes (Figure S2). Absolute bacterial and fungal abundances were previously reported in Ehau-Taumaunu and Hockett [38] and varied between 1 × 10^10^ and 9 × 10^10^ 16S rRNA copies and 1 × 10^6^ and 1 × 10^7^ ITS copies, with no general trends over the course of passaging.

We also used shotgun sequencing to explore taxonomy at species and strain levels. Only LCP and LCO treatments were used to capture the effects of the exogenous microbiome across the initial passage, the peak in disease severity, the end of the decline, and the final passage (Passage 1, 4, 6, and 9; Fig. 1—grey box). Similar to amplicon sequencing, Kraken 2 identified high bacterial relative abundance for *Pseudomonas* and *Xanthomonas* in the LCP treatment, and *Xanthomonas* for the LCO treatment. *Pseudomonas syringae* group *genomosp. 3*, represented by *Pto* DC3000, reflected the majority of the *Pseudomonas* relative abundance in both LCP and LCO (Fig. 2A, B; Figure S3). Multiple *Xanthomonas* species were identified, two of which are known to be plant pathogens of tomato. Additionally, *Microbacterium* species were present in greater abundance in LCO compared to LCP (Figure S3).

Viral sequences from the shotgun dataset were also processed using VirSorter and vConTACT2. The phages observed are likely prophages due to sample processing through a filter. A single phage, *Xanthomonas* phage Xf109, was identified across all four passages regardless of treatment. Other viral sequences were recovered at particular passages for different treatments, such as *Pseudomonas* phage MR15 at Passages 1 and 4 for LCP and Passage 6 for LCO; *Microbacterium* phage Min1 at Passage 4 in LCO; and *Arthrobacter* phage vB-ArS-ArV2 at Passage 9 in LCP.

### Taxonomic diversity of bacteria and fungi influenced by passaging and *Pto* inoculation

**Fig. 3:**
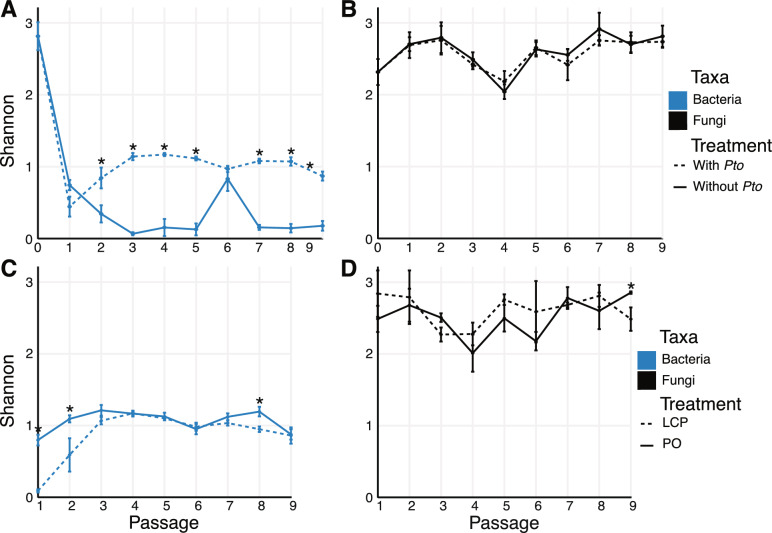
Shannon diversity was compared between treatments with *Pto* (LCP and PO) and without *Pto* (LCO and BO) and also between individual treatments, Leaf Community with Pathogen (LCP) and Pathogen only (PO), for (**A** and** C**) bacterial and (**B** and** D**) fungal ASVs. Passage 0 indicates the exogenous community sprayed at the beginning of passage 1. * P ≤ 0.05

The exogenous phyllosphere microbiomes (i.e., Passage 0) displayed similar Shannon diversity values for bacteria and fungi, but bacterial diversity was low compared to fungi from Passage 1 onwards (Fig. 3A, B). For bacterial composition, Shannon diversity after Passage 2 for treatments with *Pto* (LCP and PO) was significantly higher than treatments without *Pto* (LCO and BO), except for at Passage 6, which could be related to the high relative abundance of *Pseudomonas* (Figs. 3A; Table S2). There were no differences in fungal diversity across passages, regardless of *Pto* inoculation (Fig. 3B). When comparing LCP and PO, only Passages 1, 2, and 8 were significantly different for bacterial alpha diversity (Fig. 3C, D).

**Fig. 4:**
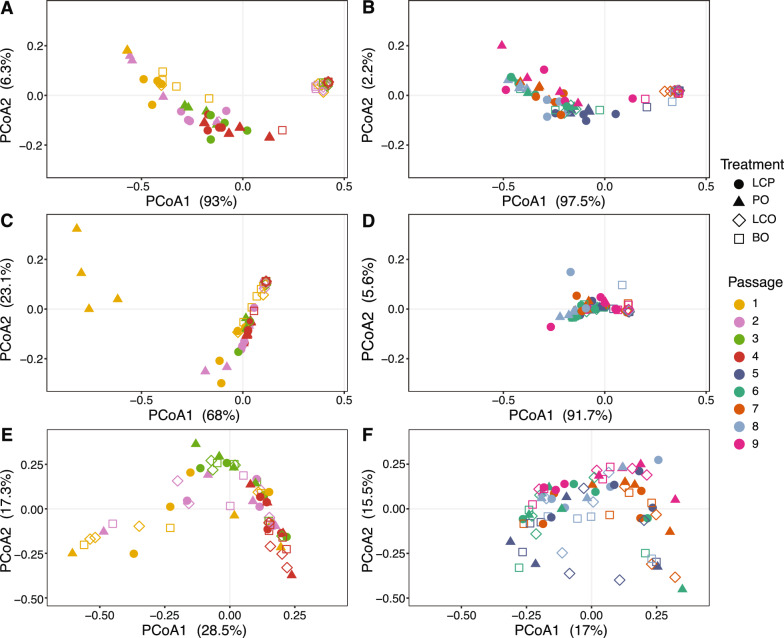
Community composition of bacterial and fungal microbiomes between passages from amplicon sequencing. Principal Coordinates Analysis (PCoA) of Bray–Curtis dissimilarity between passages 1–4 (initial until peak in disease severity) and passages 5–9 (end of peak to low disease severity) for bacteria (**A** and **B**), bacteria excluding ASV3 identified as *Pseudomonas syringae* (**C** and **D**), and fungi (**E** and** F**)

Treatments with and without *Pto* were also clustered in a PCoA ordination of bacterial composition before the peak (i.e., Passages 1–4), as well as after the peak (i.e., Passages 5–9) in disease severity (Fig. 4A, B). Variance in bacterial composition was affected by both treatment and passage, though treatment accounted for the largest amount of variation (54% during rise of disease severity and 68% during the decline; Table 1). Treatment continues to primarily explain variation when ASV3 (identified as *Pseudomonas syringae*) was excluded from bacterial composition at 33% and 55%, respectively (Fig. 4C, D; Table 1). This suggests that pathogen inoculation influenced bacterial diversity, and moreso for the passages associated with the decline in disease severity. Fungal composition resulted in no distinct clustering, with passaging playing a significant role in variation before (20%) and after the peak (15%; Figs. 4E, F; Table 1).

**Table 1:** PERMANOVA on Bray–Curtis dissimilarities across selected passages for bacterial and fungal compositions

Comparison	Microbiome	Factor	R^2^	P value
Passages 1 to 4	Bacteria	Passage	0.23494	0.001*
		Treatment	0.54433	0.001*
		Passage:Treatment	0.09304	0.001*
	Bacteria excluding AVS3	Passage	0.17134	0.001*
		Treatment	0.33702	0.001*
		Passage:Treatment	0.34248	0.001*
	Fungi	Passage	0.20420	0.001*
		Treatment	0.02128	0.999
		Passage:Treatment	0.10956	0.826
Passages 5 to 9	Bacteria	Passage	0.10682	0.001*
		Treatment	0.68066	0.001*
		Passage:Treatment	0.07144	0.009*
	Bacteria excluding AVS3	Passage	0.10841	0.001*
		Treatment	0.55168	0.001*
		Passage:Treatment	0.08827	0.056
	Fungi	Passage	0.14618	0.001*
		Treatment	0.04525	0.079
		Passage:Treatment	0.14900	0.234

R^2^ values represent the percentage of bacterial community variation between the selected passages explained by indicated experimental factors

^a^P-value FDR adjusted across all passages

*Indicates statistical significance (P ≤ 0.05)

An exogenous phyllosphere community applied to tomato plants before passaging did not significantly influence bacterial or fungal composition. Treatments that resulted in plant disease suppression, LCP and PO, showed no clustering for bacterial and fungal composition (Figure S4A and S4B). In a different comparison, the LCP treatment clustered more densely together than LCO for bacteria, with a treatment effect of 66% on compositional variation (Figure S4C; Table S3). Fungal composition showed no clustering between LCP and LCO, and no significant treatment effect (Figure S4D, Table S3). These results suggest that the pathogen inoculum was a major factor affecting bacterial composition across passaging, yet the exogenous phyllosphere microbiome did not influence bacterial or fungal composition.

To potentially identify key players in the transition from a disease-conducive to a disease-suppressive microbiome, we compared the composition from early and late passages (Passage 1 vs 9) and what may have changed between the critical transition from peak in disease to low disease (Passage 4 vs 7). Bacterial diversity of treatments with and without *Pto* from Passage 1 and 9 typically clustered together regardless of passage, suggesting similarity in taxa at the beginning and end of the experiment (Figure S5A). The composition of treatments with and without *Pto* between Passages 4 and 7 was significantly different and accounted for 89% of the variation (P = 0.01), yet differences between passages were smaller (24%) despite differing levels in disease severity (Figure S5B). When ASV3 was excluded from the bacterial dataset, PO clustered away from the other treatments between Passages 1 and 9, whereas all samples clustered closely together for Passages 4 and 7 (Figure S5C and S5D). For fungi, beta diversity showed significant clustering based on passage only (Figure S5E and S5F; Table S3).

### Several bacterial ASVs differed in abundance between treatments with and without *Pto* and were influenced by the presence of ASV3 (*Pseudomonas syringae*)

**Fig. 5:**
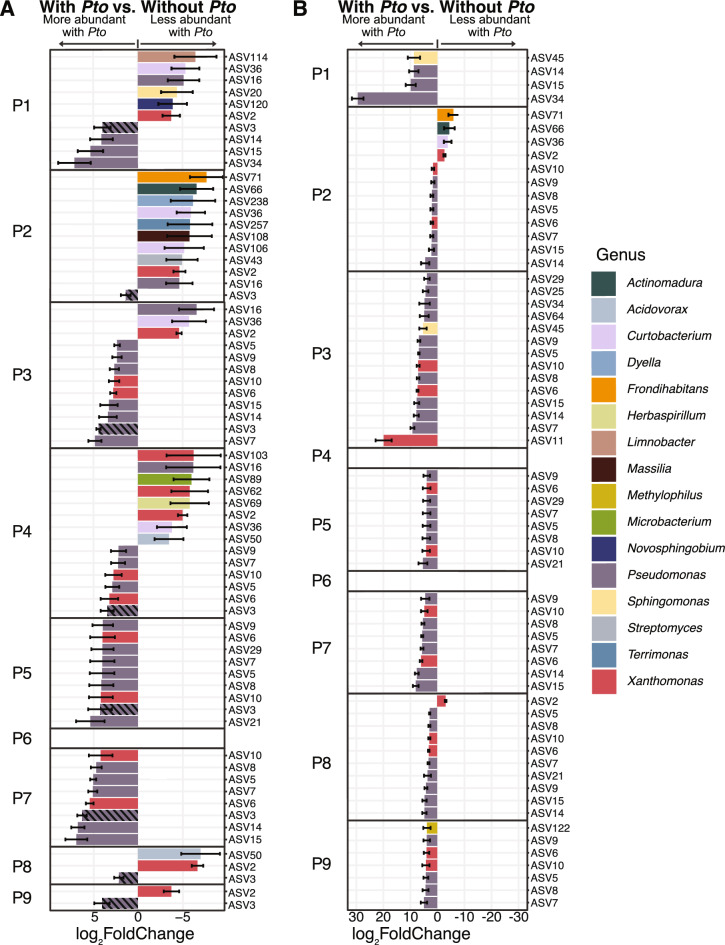
Differential abundance of bacterial ASVs between treatments with *Pto* (LCP and PO) and without *Pto* (LCO and BO). Positive and negative log_2_ fold changes identified in two or more methods (DESeq2; ANCOM; Corncob; Limma Voom) indicate significantly increased and decreased abundances (Benjamini–Hochberg-adjusted P(FDR) ≤ 0.05) for (**A**) all ASVs and (**B**) ASVs excluding ASV3 identified as *Pseudomonas syringae*. P1 to P9 indicate passage numbers. Bars with diagonal shading are representing ASV3. Error bars represent standard errors. Bars are colored by genus

Differences were observed when examining the differential abundance of bacterial ASVs between treatments with or without *Pto* across passages. Several ASVs assigned to *Pseudomonas* and *Xanthomonas* were significantly more abundant in treatments with *Pto* across multiple passages (Fig. 5A). For example, ASV14, identified as *Pseudomonas antarctica,* was more abundant during early and late passages. ASV6 and ASV10, identified as *Xanthomonas,* were less abundant across several passages. A single ASV, ASV3 assigned as *Pseudomonas syringae*, remained more abundant at every passage in treatments with *Pto* than those without *Pto* (Fig. 5A). No ASVs differed significantly in abundance between treatments at Passage 6, likely due to the high relative abundance of *Pseudomonas* in treatments without *Pto*.

These results between treatments suggest that shifts in abundance might be primarily driven by the exogenous addition of *Pseudomonas syringae* pv. *tomato* into LCP and PO, so we repeated the differential abundance analyses for bacteria with ASV3 excluded. The number of taxa that were less abundant in treatments with *Pto* compared to those without *Pto* decreased across all passages (Fig. 5B). For early passages, many differentially abundant taxa remained as observed in previous analyses with ASV3 present, yet many new ASVs became more abundant in treatments with *Pto*. Passages 5 and 7 remained unchanged in the ASVs that were differentially abundant, with small differences in the log2 fold change. The later passages had a large number of ASVs that were more abundant in *Pto* containing treatments when AVS3 was masked from the analysis. The majority of these ASVs were assigned to *Pseudomonas* and *Xanthomonas* (Fig. 5B). No ASVs were differentially abundant for treatments with *Pto* for Passages 4 and 6. The removal of ASV3 appears to affect passages differently, where ASVs in some passages were reduced by the high abundance of ASV3 and others were not affected by its presence.

Differentially abundant fungal ASVs for treatments with *Pto* in general were more variable compared to bacteria (Figure S6). Only a single taxa showed a consistent pattern of differential abundance. *Epicoccum dendrobii* (ASV9) was more abundant with *Pto* compared to treatments without *Pto* at Passages 2, 3, and 6. Overall, the stochasticity in the abundance of different fungal ASVs continues to suggest that *Pto* inoculation did not have a strong influence on fungal composition.

### Antagonistic functional categories were more abundant in the leaf community with *Pto* and *Pseudomonas* predicted to play a key role in the functional potential of the microbiome

The metagenomic sequences of LCP and LCO clustered in a PCoA ordination of bacterial composition based on treatment similar to the amplicon samples (Figure S7). To examine the differences in functional potential of the LCP and LCO phyllosphere communities across the four passages, we used EggNOGmapperv2 to obtain annotations derived from Pfam protein domain predictions (Table S1). The functional potential of each treatment at Passages 1, 4, 6, and 9 consisted of three datasets for comparison: (1) all contigs, (2) all contigs excluding contigs identified to the *Pseudomonas* genus, and (3) all contigs excluding those classified as *Pseudomonas syringae* or unclassified. Between 70 and 90% of nonredundant genes annotated in EggNOG for all datasets could be aligned to Pfam IDs (Table S4). We selected genes involved in microbial antagonism that could be associated with disease suppression and summed the relative gene abundance within multiple functional categories.

**Fig. 6:**
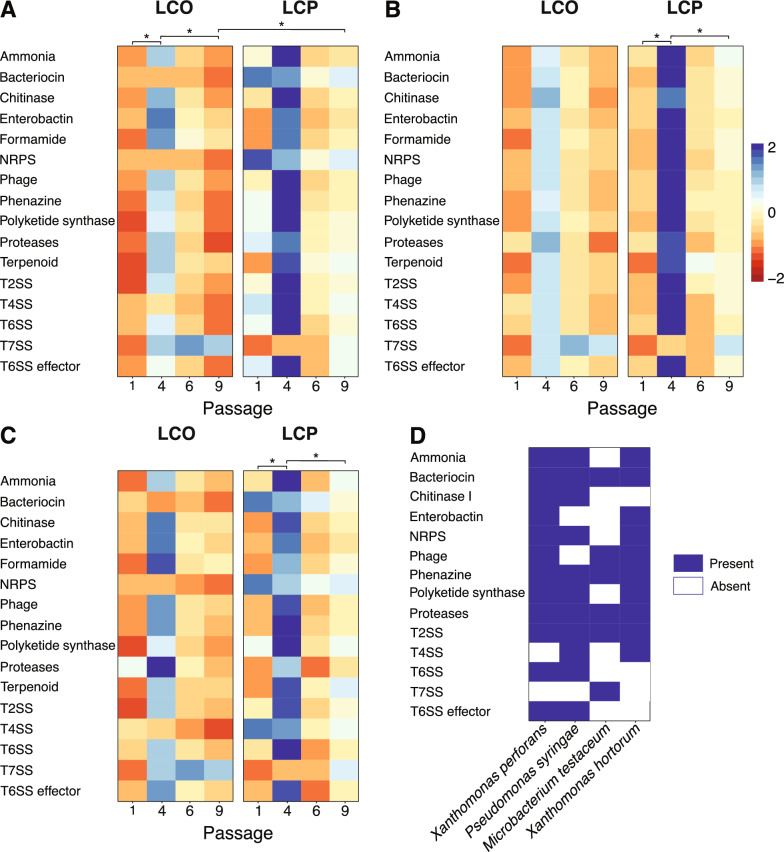
The relative abundance of different functional categories associated with microbial antagonism. Annotated genes with known antagonism function were selected based on Pfam ID and grouped into categories. The relative abundance of each gene was expressed as TPM (transcripts per million) estimated by CoverM. The heatmaps for (**A**) all contigs, (**B**) no *Pseudomonas* assigned contigs, and (**C**) no *Pseudomonas syringae* and unassigned contigs from summed TPM values for each functional category. Blue indicates high abundance and red for low abundance compared to the column mean. (**D**) A presence/absence matrix of functional categories within identified MAGs. Specific Pfam IDs used for the functional categories can be found in Table S4. *P ≤ 0.05

Analyses showed a higher abundance of functional categories related to antagonism at the peak of disease (i.e., Passage 4) across all datasets and treatments (Fig. 6). When all contigs are present (dataset 1) and treatments are considered across all passages, then the treatment effect between LCP and LCO in a one-way ANOVA with a post-hoc Tukey HSD is significant (P = 0.007; Fig. 6A). Of interest, there was a significant treatment difference at Passage 9 (P = 0.05) indicating differentiation of the disease suppressive microbiome that formed at the end of the experiment. When we examine each treatment separately, the passage effect is significant between several passages of the treatment with *Pto*, LCP (Fig. 6A). The initial and final LCP passages (Passage 1 and 9) where disease severity were at their lowest were significantly different to Passage 4 where the disease was at its peak (P = 0.038 and P = 0.025, respectfully). Whereas, there were no significant differences between the passages of treatment without *Pto* (i.e., LCO). Although LCP Passage 4 showed a high abundance in the heatmap, a pairwise comparison to LCO Passage 4 was not significantly different.

When we excluded the contigs assigned to *Pseudomonas* (dataset 2) or *Pseudomonas syringae* and unassigned contigs (dataset 3), we observed a similar abundance pattern for the antagonistic functional categories compared to all contigs (Fig. 6B, C). Both datasets presented high gene abundance at Passage 4 within LCP and LCO treatments, however only LCP displayed significant differences to Passage 1 (P = 0.026 and P = 0.003) and Passage 9 (P = 0.020 and P = 0.007). Yet, there were no pairwise differences between LCP and LCO at Passages 4 and 9, suggesting the removal of the contigs associated with *Pseudomonas* or that were unassigned did not change the overall antagonistic functional potential.

### MAGs show that dominant taxa provide key antagonistic functions

Metagenomic assembled genomes (MAGs) were constructed from the shotgun sequencing data from each sample. A total of 20 MAGs from the four passages and LCP and LCO treatments were dereplicated, producing four unique representative MAGs with a minimum 70% completeness and < 5% contamination. The Genome Taxonomy Database Toolkit (GTDB-Tk) identified the four unique MAGs: *Microbacterium testaceum*, *Pseudomonas syringae*, *Xanthomonas hortorum*, and *X. perforans* (Fig. 6D). Of the 21 broad functional categories, bacterioferritin, formamide, terpene, and terpenoids were not present in any of the MAGs. In a presence/absence matrix, bacteriocins, phenazine, proteases, and T2SS were present across all four MAGs (Fig. 6D). MAGs identified to the *Pseudomonas* and *Xanthomonas* genera had the highest number of functional categories covering key antagonistic traits known to both genera. The MAG closely identified as *Microbacterium testaceum* only had Pfam IDs for six of the functional categories (Fig. 6D).

## Discussion

Disease-suppressive phyllosphere microbiomes differentiated from non-suppressive microbiomes in both composition and activity due to the influence of *Pto*. Passaging communities between tomato plants led to multiple differentially abundant taxa, with *Pseudomonas* and *Xanthomonas* enriched in suppressive microbiomes, and only *Xanthomonas* in the conducive microbiomes. The exploration of several functional genes showed an increase in the antagonistic trait-encoding potential in the suppressive microbiomes compared to microbiomes conducive to disease. Thus, based on the overall trends associated with bacteria compared to fungi and the additional ability to the cryogenically store and resuscitate these passaged communities [38], the development of disease suppressive communities to bacterial speck of tomato is likely due to the contributing bacterial fraction.

Bacterial and fungal composition across all passages and treatments was dominated by a few highly abundant genera. The low bacterial diversity differs from the high diversity that has been observed in some suppressive soils [18, 21] (Mazzola and Gu 2007), suggesting that disease suppression in the phyllosphere, at least in this context, could be attributed to a few antagonistic microorganisms. *Pseudomonas* was highly abundant for treatments with *Pto* (LCP and PO) and was similar to the relative abundance of *Pseudomonas syringae* group genomospecies 3, which includes *Pto* and several other pathogenic *P. syringae* [78–80]. In the absence of ASV3, the relative abundance of *Pseudomonas* largely decreased, indicating that *Pseudomonas syringae* was the dominant species. Unexpectedly, the relative abundance of *Pseudomonas* decreased when disease severity and recoverable *Pto* populations were observed at their highest [38]. We hypothesize that this discrepancy is likely due to abundant dead Pto cells from previous passages being carried over to subsequent passages. Additionally, when the relative abudance of *Pseudomonas* decreased, *Xanthomonas* increased, even though this genus is typically not dominant in the phyllosphere [81]. This indicates that passaging could also be enriching for *Xanthomonas* under these experimental conditions, including *Xanthomonas perforans* a causal agent of bacterial spot of tomato [82–85]. Yet, minimal to no bacterial spot symptoms were observed. Therefore, this experimental method may lead to microbiome states that might not be found in the field and that enable the development of disease-suppressive microbiomes.

The addition of an exogenous community mattered less than the background microorganisms (derived from the greenhouse environment) for bacteria, whereas, for fungi, this was not the case. Our community data shows that exogenous field-collected microbiome did not provide additional taxa beyond the background level natively present in the greenhouse environment. Multiple prophages were also identified that could target highly abundant bacteria, but further investigation into the viral diversity and abundance is needed, since our sampling approach did not enrich the viral fraction of the microbiome, as others have done [86].

Bacterial alpha and beta diversity were heavily influenced by the presence and absence of *Pto*, which was not the case for fungi. Bacterial evenness of all ASVs was lower for samples with *Pto* (LCP and PO) compared to samples without *Pto* (LCO and BO), yet remained even lower than fungi from Passages 1 to 9. General clustering of disease-suppressive or disease-conducive samples in a PCoA was also observed for bacteria across all passages and between key passage comparisons. However, in the absence of ASV3, there was no clustering based on treatment. This suggests that successive passaging with the phytopathogen was a key driver in potentially enhancing disease suppression [14]. The alpha and beta diversity of fungi were not significantly affected by treatment or the interaction between passage and treatment, indicating that fungal composition was stochastic regardless of *Pto* introduction. Aboveground fungal composition on plants affected by *Fusarium* wilt disease, caused by the *Fusarium oxysporum* species complex, were less stable and directly impacted by the phytopathogen than the bacterial communities [87]. The large skew in the bacteria:fungi ratio where the majority of fungal ASVs were rare within samples also supports the lack of fungal involvement. Therefore, disease suppression in the phyllosphere communities could be due more to leaf-associated bacteria than fungi.

The impact of *Pto* was evident in the shifts of relative abundance of bacteria, especially in the absence of ASV3, supporting our hypothesis that *Pto* would have an influence on microbial diversity. In comparison to analyses with all ASVs, multiple and different *Pseudomonas* ASVs remained more abundant in treatments with *Pto*, indicating the potential of several bacterial species or sub-species involved in disease suppression initially hidden by the high abundance of ASV3. A minority of differentially abundant ASVs were also less abundant in treatments with *Pto* compared to when ASV3 was present in the analyses, most of which represented other bacterial taxa. Additionally, assuming that ASV3 acts as a representative of *Pto*, its high relative abundance was observed within each passage. ASV3 was also more abundant at Passage 9, suggesting that *Pto* was still present in the disease-suppressive communities similar to the high inoculum levels observed in Take-all decline soils [15, 88]. The influence of *Pto*, however, is unknown for fungal relative abundance with a small number of ASVs being differentially abundant across several passages.

To further understand disease-induced changes in the LCP (leaf community with *Pto*) microbiomes, the functional potential for microbial antagonism was investigated at key passages between disease-suppressive and non-disease-suppresive microbiomes. Metagenomic analyses of annotated genes by Pfam indicated that many functional genes involved in antagonism, such as bacteriocins, toxins, and secondary metabolites were abundant [89–93]. The limited number of *Pseudomonas*, *Microbacterium*, and *Xanthomonas* MAGs from replicate microbiomes is typical for such samples [4, 94, 95] and shows a likely contribution to the relative abundance of several functional categories. There was a statistical difference between the functional potential of LCP and LCO microbiomes at the end of the passaging where disease severity remained low. This suggests the relative abundance of genes related to antagonism was higher for a developed disease-suppressive microbiome and could be enabling continual disease suppression as observed in Ehau-Taumaunu and Hockett [38]. The treatment difference was negligible when the dataset was altered to reduce the influence of *Pseudomonas*, *Pseudomonas syringae,* and unidentified contigs (including the phytopathogen), with a decrease in the relative abundance of several functional categories in Passages 1, 6, and 9, suggesting that *Pseudomonas* may be providing a high abundance of antagonism related genes. The functional potential of microbiomes was generally highest at the peak in disease (Passage 4) across the three datasets, indicating the possibility of intra- and interspecies antagonism towards *Pto*. However, in the absence of unidentified contigs there was no differences between LCP and LCO functional potential at passage 9, suggesting the presence of *Pseudomonas* species may be necessary for disease suppression.

## Conclusions

The present study investigated the taxonomic and functional potential of several passaged communities that had previously shown disease suppression to bacterial speck in tomato through an experimental passaging design [38]. Our sequencing findings demonstrate that disease-suppressive microbiomes had low bacterial diversity and that the phytopathogen *Pto* caused shifts in differentially abundant bacterial taxa, whereas the fungal microbiomes generally remained the same. As microbiomes developed over several passages, antagonistic genes increased during high disease severity and generally were more relatively abundant in disease-suppressive microbiomes. Our work has set a foundation for further testing and understanding of the contributions of taxa to disease suppression we observed against *Pseudmonas syringae* pv. *tomato* in tomato. Additional testing of the experimental design and greater taxonomic identification and annotation would verify the utility of this approach within the tomato-*P. syringae* system and other plant-pathogen systems. Additionally, transcriptomics on the interactions between pathogens, antagonists, and the plant could provide insight into gene expression and modes of actions of microorganisms.

## Supplementary Information

Additional file1 (PDF 544 KB)

## Data Availability

The dataset(s) supporting the conclusions of this article are available in the NCBI Sequence Read Archive database under BioProject ID PRJNA932145.

## Abbreviations

ANI: Average nucleotide identity
ANOVA: Analysis of variance
ASV: Amplicon sequencing variant
DNA: Deoxyribonucleic acid
FDR: False discovery rate
ITS: Internal transcribed spacer
MAGs: Metagenomic assembled genomes
PCoA: Principle coordinate analysis
PERMANOVA: Permutational analysis of variance
RNA: Ribonucleic acid
TPM: Transcripts per million
