# Supplementary material for: Rapid and sustained differentiation of disease-suppressive phyllosphere microbiomes in tomato following experimental microbiome selection
Source: Environ Microbiome. 2025 Jul 1;20:77. doi: 10.1186/s40793-025-00734-1 (PMC12211302; doi:10.1186/s40793-025-00734-1)
Supplement: Supplementary file 1 — Additional file1 (PDF 544 KB) [file 40793_2025_734_MOESM1_ESM.pdf]

## Supplementary Material

**Supporting information related to the manuscript: Rapid and sustained differentiation of disease-suppressive phyllosphere microbiomes in tomato following experimental microbiome selection**

Hanareia Ehau-Taumaunu[1], Terrence H. Bell[2], Javad Sadeghi[2] and Kevin L. Hockett[1,3,4*]

[1]Department of Plant Pathology and Environmental Microbiology, The Pennsylvania State University, University Park, PA 16802, U.S.A.

[2]Department of Physical and Environmental Sciences, University of Toronto – Scarborough, Toronto, ON, Canada

[3]Center for Infectious Diseases Dynamics, The Pennsylvania State University, University Park, PA 16802, U.S.A.

[4]The Huck Institutes of the Life Sciences, The Pennsylvania State University, University Park, PA 16802, U.S.A.

*Corresponding author: Kevin Hockett; E-mail: klh450@psu.edu

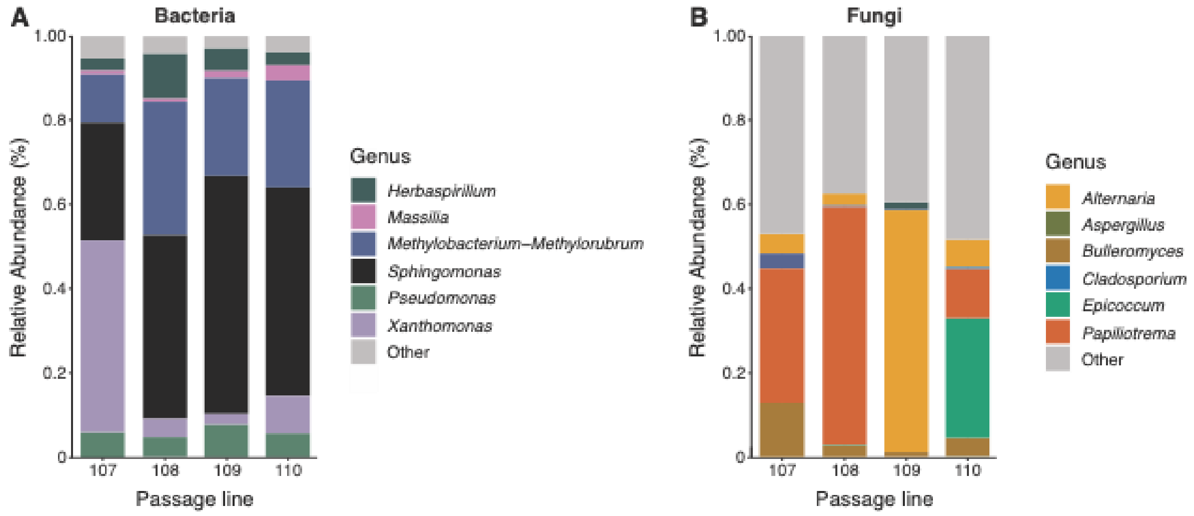


**Figure S1.** Source composition at the genus level across four passage lines (107, 108, 109, 110) for each treatment. ‘Other’ includes all taxa assigned <3%.

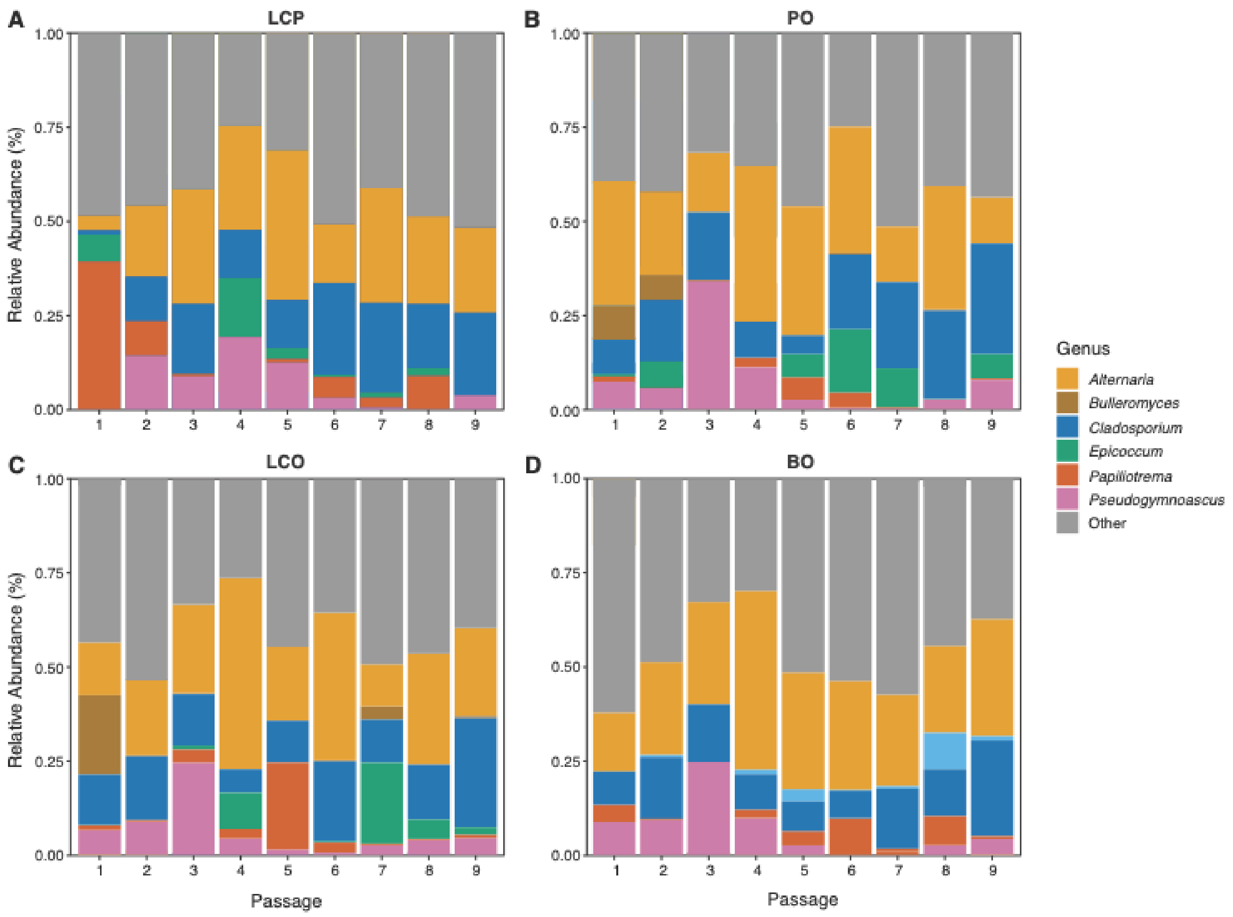


**Figure S2.** Fungal composition at the genus level across treatments across passages 1 to 9. The relative abundance (%) presented for genera >3%. 'Other' includes all taxa assigned <3%.

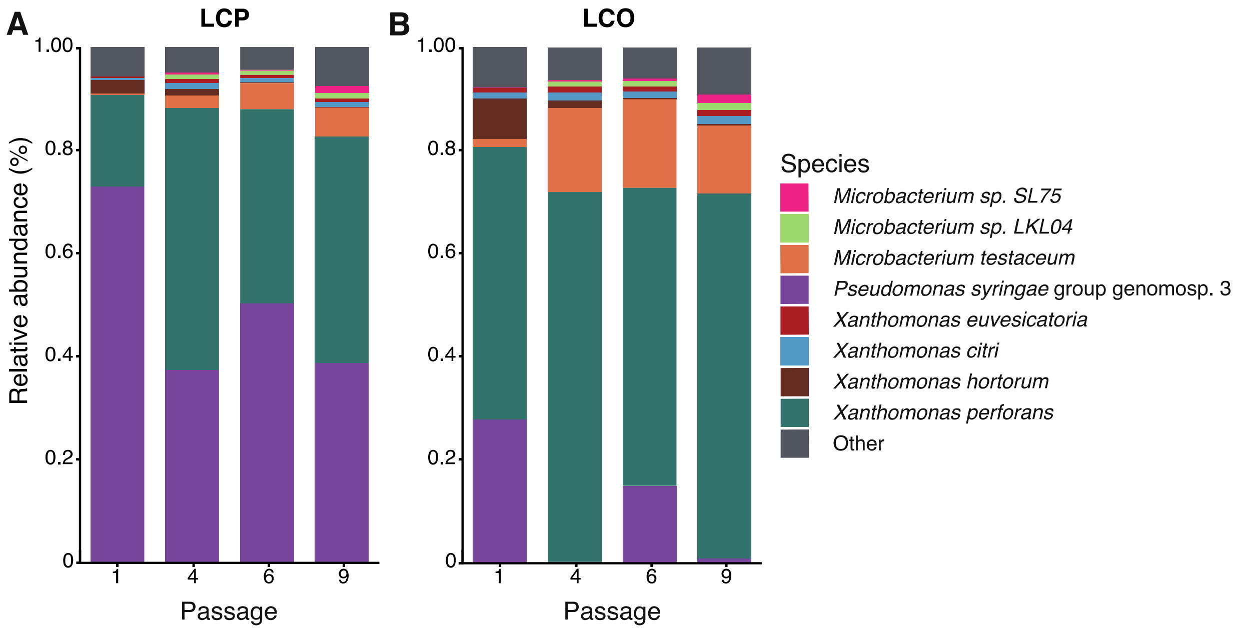


**Figure S3.** Species relative abundance for (**A**) Leaf community with *Pto* (LCP) and (**B**) Leaf community only (LCO) microbiomes. Dominant genera assigned and estimated using Kraken 2 at passages 1, 4, 6, and 9 representing different stages of disease progression throughout the passaging experiment. 'Other' includes all taxa assigned <3%.

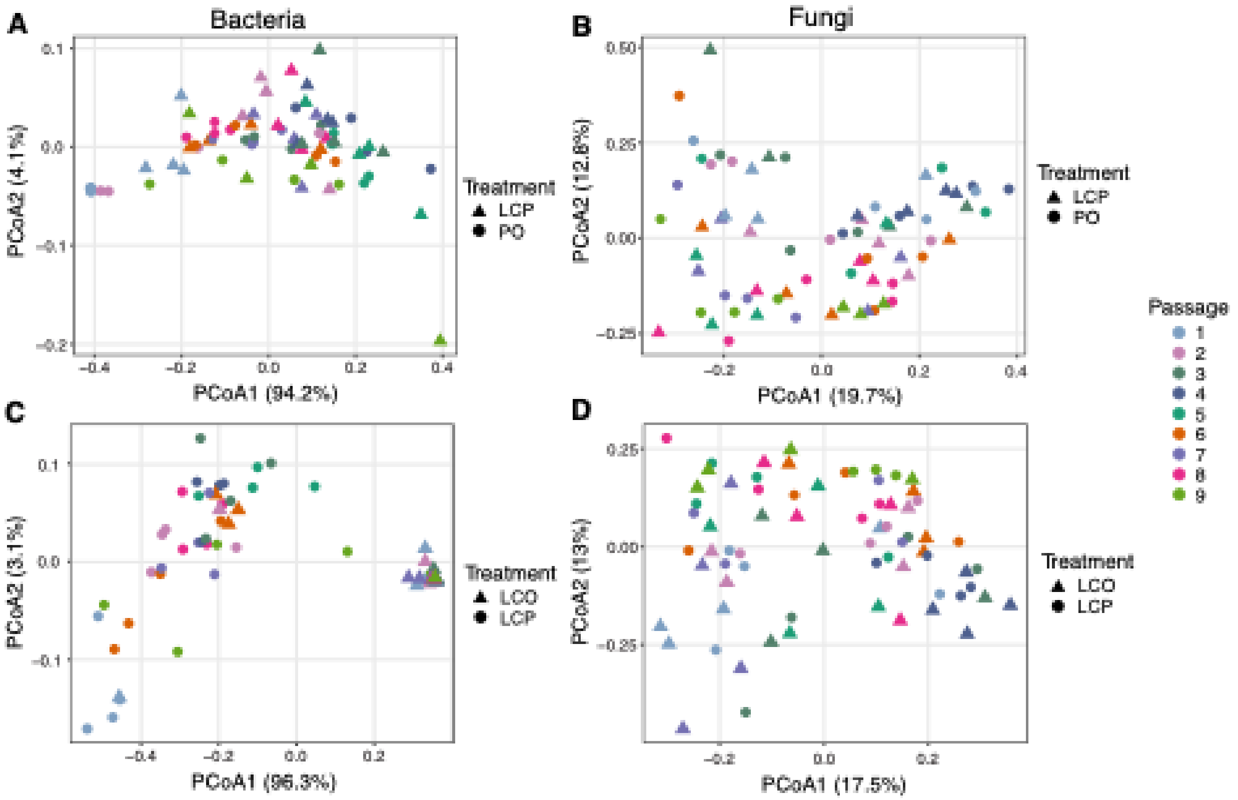


**Figure S4.** Principal coordinates analysis (PCoA) presented the distribution of LCP vs PO treatments for (**A**) bacterial and (**B**) fungal microbiomes, along with LCO vs LCP treatments for (**C**) bacterial and (**D**) fungal microbiomes. * P≤0.05.

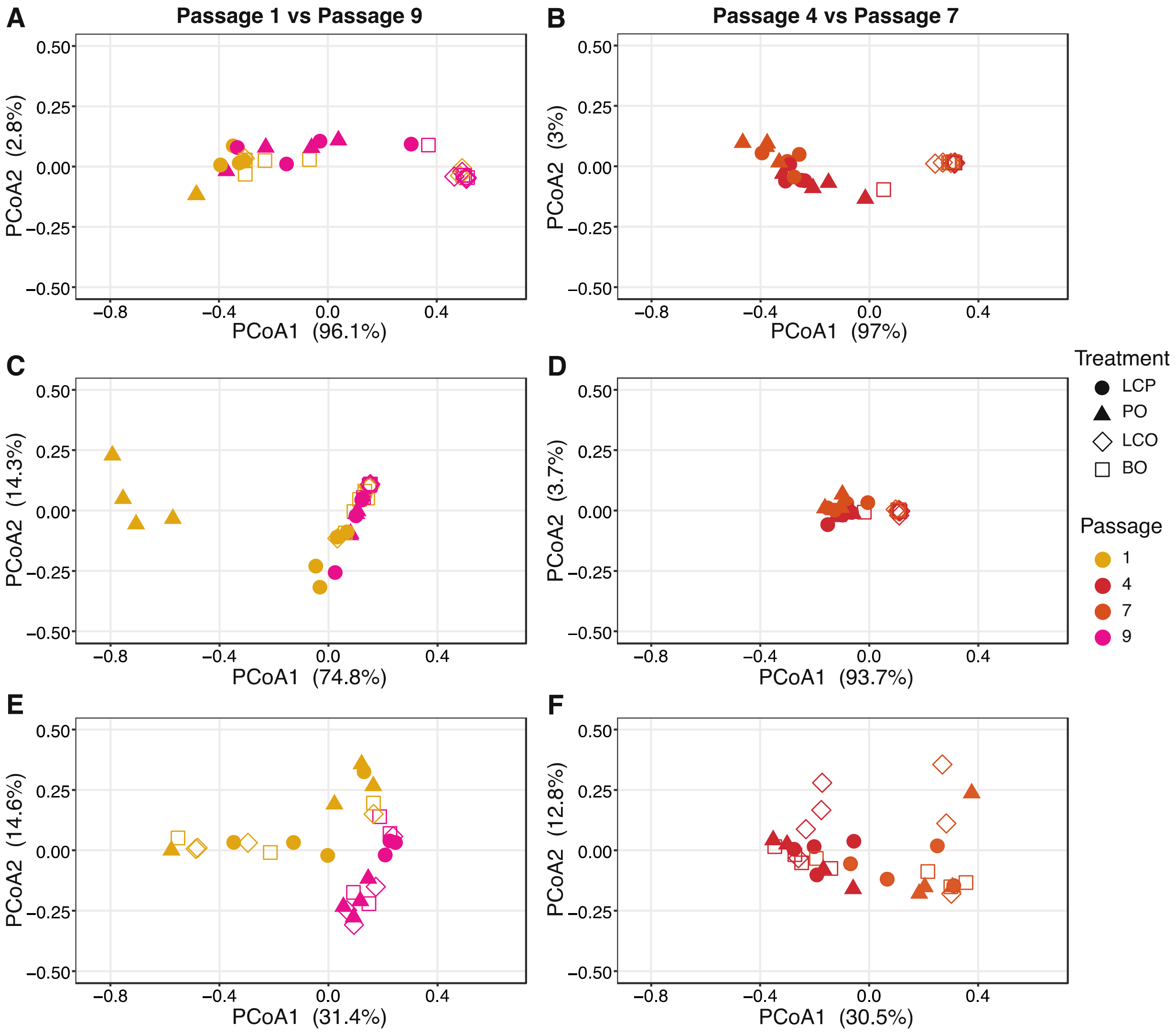


**Figure S5.** Similarity of microbiomes for bacteria and fungi at key passages related to disease severity. Principal Coordinates Analysis (PCoA) of Bray-Curtis dissimilarity between passages 1 and 9 (low disease before and after disease peak) or passages 4 and 7 (peak and end of peak disease severity) for bacteria (**A** and **B**), bacteria excluding ASV3 identified as *Pseudomonas syringae* (**C** and **D**), and fungi (**E** and **F**).

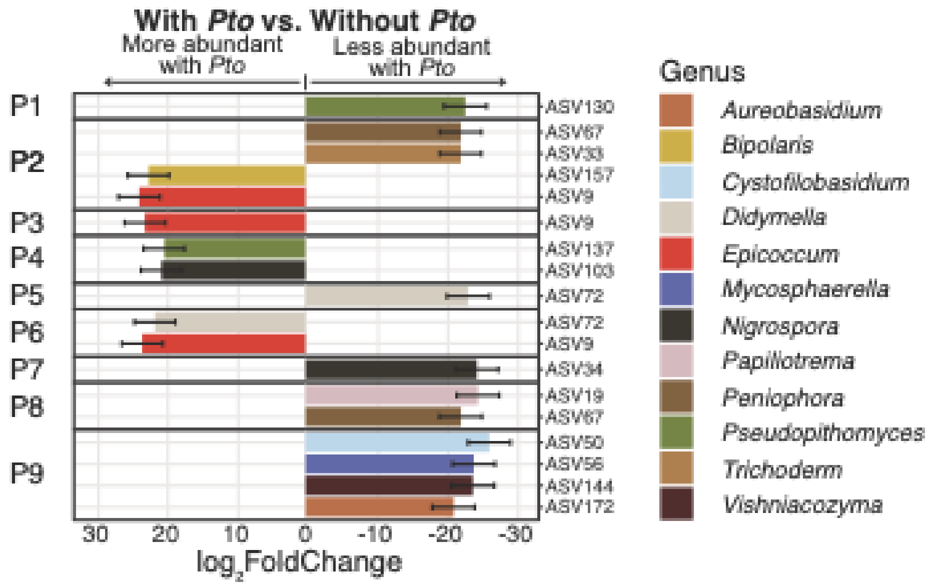


**Figure S6.** Differentially abundant fungal genera from amplicon sequencing. Significant $log_2$ fold change of different fungal genera between treatments with *Pto* (LCP and PO) and without *Pto* (LCO and BO) across passages (P≤0.05). Error bars represent standard errors. Bars are coloured by genera.

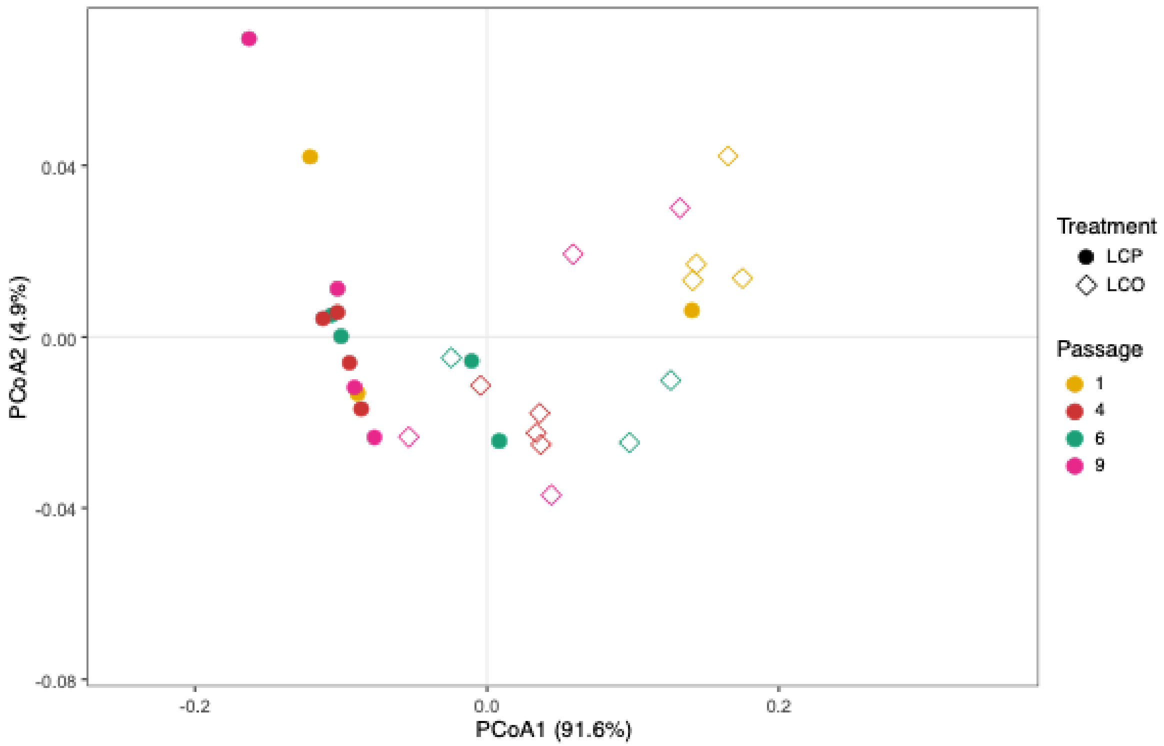


**Figure S7.** Principal coordinates analysis (PCoA) of Bray-Curtis dissimilarity between LCP and LCO microbiomes from metagenomic sequencing. * P≤0.05.

**Table S1.** Summary of information from amplicon and metagenomic sequencing

| Amplicon | | Total reads | Range of reads after quality filtering | Mean number of reads per sample after quality filtering | |
|---|---|---|---|---|---|
| Bacteria | | 3693727 | 11448 - 38770 | 21290 | |
| Fungi | | 3460068 | 3526 - 35750 | 9012 | |
| Metagenomic | | Mean number of reads | Mean number of reads after quality filtering | Mean number of contigs | Number of PFAM IDs |
| LCP | P1 | 3944803 | 3210461 | 8246 | 12969 |
| | P4 | 4375087 | 3506557 | 7842 | 14272 |
| | P6 | 4911585 | 3644367 | 6845 | 13306 |
| | P9 | 4589054 | 3415975 | 9203 | 14802 |
| LCO | P1 | 4586201 | 2745397 | 15550 | 14992 |
| | P4 | 4360172 | 3124946 | 14198 | 10779 |
| | P6 | 4239668 | 2890412 | 14198 | 16458 |
| | P9 | 4337250 | 2518476 | 32507 | 13165 |

**Table S2.** Shannon diversity statistics compared between treatments with *Pto* (LCP and PO) and without *Pto* (LCO and BO) using the Kruskal–Wallis test.

| Passage | Bacteria | Fungi |
|---|---|---|
| 1 | 0.21375 | 0.96000 |
| 2 | 0.12857 | 0.96000 |
| 3 | 0.00144* | 0.96000 |
| 4 | 0.00036* | 0.96000 |
| 5 | 0.00036* | 0.96000 |
| 6 | 0.65000 | 0.96000 |
| 7 | 0.00036* | 0.96000 |
| 8 | 0.00036* | 0.96000 |
| 9 | 0.00056* | 0.96000 |

[a] P-value FDR adjusted across all passages

* Indicates statistical significance ($P \leq 0.05$)

**Table S3.** PERMANOVA on Bray-Curtis dissimilarities of bacterial and fungal microbiomes between selected passages and treatments in supplemental.

| Comparison | Microbiome | Factor | $R^2$ | P value |
|---|---|---|---|---|
| **Passages 1 vs 9** | Bacteria | Passage | 0.24240 | 0.001* |
| | | Treatment | 0.50968 | 0.001* |
| | | Passage:Treatment | 0.06772 | 0.052 |
| | Bacteria excluding AVS3 | Passage | 0.18811 | 0.001* |
| | | Treatment | 0.07277 | 0.712 |
| | | Passage:Treatment | 0.09862 | 0.284 |
| | Fungi | Passage | 0.18811 | 0.001* |
| | | Treatment | 0.07277 | 0.707 |
| | | Passage:Treatment | 0.09862 | 0.275 |
| **Passages 4 vs 7** | Bacteria | Passage | 0.23896 | 0.005* |
| | | Treatment | 0.89337 | 0.001* |
| | | Passage:Treatment | 0.03425 | 0.010* |
| | Bacteria excluding AVS3 | Passage | 0.01566 | 0.081 |
| | | Treatment | 0.84129 | 0.001* |
| | | Passage:Treatment | 0.01949 | 0.315 |
| | Fungi | Passage | 0.10841 | 0.001* |
| | | Treatment | 0.09800 | 0.141 |
| | | Passage:Treatment | 0.05875 | 0.801 |
| LCP vs PO | Bacteria | Treatment | 0.05239 | 0.004* |
| | Fungi | Treatment | 0.00901 | 0.762 |
| LCO vs LCO | Bacteria | Treatment | 0.66285 | 0.001* |
| | Fungi | Treatment | 0.01282 | 0.311 |

[a] P-value FDR adjusted across all passages

* Indicates statistical significance (P≤0.05)

**Table S4.** Pfam IDs selected for each functional category from Eggnogmapper2.

| Functional category | Pfam ID |
|---|---|
| Ammonia | Amidase<br>AstB |
| Bacteriocins | Colicin_M<br>Colicin_Pyocin<br>Colicin_V<br>CreD<br>Pyocin_S<br>PyocinActivator |
| Bacterioferritin | Ferritin |
| Chitinase I | Glyco_hydro_19 |
| Formamide | Arginase |
| Enterobactin | 4HBT |
| Non-ribosomal peptide synthase (NRPS) | AMP-binding,AMP-binding_C,Condensation,PP-binding, Thioesterase |
| Phage | Collar<br>DUF3693<br>Gp49<br>HDPD<br>Lambda_tail_I<br>P2_Phage_GpR<br>Phage_AlpA<br>Phage_base_V<br>Phage_cap<br>Phage_capsid<br>Phage_connect_1<br>Phage_CP76<br>Phage_fiber_2<br>Phage_GPD<br>Phage_GPL<br>Phage_GPO<br>Phage_holin<br>Phage_H_T_join<br>Phage_P2_GpU<br>Phage_portal<br>Phage_sheath_1,Phage_sheath_1C<br>Phage_TAC_7<br>Phage_tail<br>Phage_term_smal<br>Phage_TTP_11<br>Phage_tube<br>PhageMin_Tail<br>NfrA_C<br>Tail_P2_I<br>Tail_tube<br>Tape_meas_lam_C |
| Phenazine | PhzC-PhzF |
| Polyketide synthases | Ketoacyl-synt<br>Ketoacyl-synt_2 |
| Proteases | ClpB_D2-small<br>CLP_protease<br>Clp_N<br>Peptidase_C1_2<br>gag-asp_proteas<br>PA<br>Peptidase_S8<br>PrsW-protease<br>TerD,Trypsin_2 |
| Siderophore production | FhuF,IucA_IucC<br>SIP<br>TonB_dep_Rec<br>ABC_membrane,ABC_tran |
| Terpene | Terpene_synth |
| Terpenoids | Prenyltransf |

| | |
|---|---|
| Type II secretion system | T2SS<br>T2SSC<br>T2SSE<br>T2SSF<br>T2SSJ<br>T2SSL<br>T2SSK<br>T2SSM<br>T2SSN |
| Type IV secretion system | T4SS<br>T4BSS_DotI_IcmL<br>T4BSS_DotH_IcmK |
| Type VI secretion system | T6SS_TssF<br>T6SS_TssG<br>T6SS_VasE<br>T6SS_VipA<br>T6SS-SciN |
| Type VI secretion effectors | T6SS_HCP |
| Type VII secretion system | T7SS_ESX1_EccB |
